# Engineered Carbonic Anhydrase VI-Mimic Enzyme Switched the Structure and Affinities of Inhibitors

**DOI:** 10.1038/s41598-019-49094-0

**Published:** 2019-09-03

**Authors:** Justina Kazokaitė, Visvaldas Kairys, Joana Smirnovienė, Alexey Smirnov, Elena Manakova, Martti Tolvanen, Seppo Parkkila, Daumantas Matulis

**Affiliations:** 1 Department of Biothermodynamics and Drug Design, Institute of Biotechnology, Vilnius University, Saulėtekio 7, Vilnius, LT-10257 Lithuania; 2 Department of Bioinformatics, Institute of Biotechnology, Vilnius University, Saulėtekio 7, Vilnius, LT-10257 Lithuania; 3 Department of Protein-DNA Interactions, Institute of Biotechnology, Vilnius University, Saulėtekio 7, Vilnius, LT-10257 Lithuania; 4 Department of Information Technology, University of Turku, FI-20520 Turku, Finland; 5 Tampere University, Faculty of Medicine and Health Technology; Fimlab Ltd, Tampere University Hospital, Arvo Ylpön katu 34, FI-33520 Tampere, Finland; 6 Present Address: Division of Biochemistry, the Netherlands Cancer Institute, Amsterdam, the Netherlands

**Keywords:** Enzymes, Medicinal chemistry

## Abstract

Secretory human carbonic anhydrase VI (CA VI) has emerged as a potential drug target due to its role in pathological states, such as excess acidity-caused dental caries and injuries of gastric epithelium. Currently, there are no available CA VI-selective inhibitors or crystallographic structures of inhibitors bound to CA VI. The present study focuses on the site-directed CA II mutant mimicking the active site of CA VI for inhibitor screening. The interactions between CA VI-mimic and a series of benzenesulfonamides were evaluated by fluorescent thermal shift assay, stopped-flow CO_2_ hydration assay, isothermal titration calorimetry, and X-ray crystallography. Kinetic parameters showed that A65T, N67Q, F130Y, V134Q, L203T mutations did not influence catalytic properties of CA II, but inhibitor affinities resembled CA VI, exhibiting up to 0.16 nM intrinsic affinity for CA VI-mimic. Structurally, binding site of CA VI-mimic was found to be similar to CA VI. The ligand interactions with mutated side chains observed in three crystallographic structures allowed to rationalize observed variation of binding modes and experimental binding affinities to CA VI. This integrative set of kinetic, thermodynamic, and structural data revealed CA VI-mimic as a useful model to design CA VI-specific inhibitors which could be beneficial for novel therapeutic applications.

## Introduction

Human carbonic anhydrases (CAs) are widespread enzymes known for over 80 years^1^. CAs regulate both intracellular and extracellular pH homeostasis through the catalysis of reversible carbon dioxide hydration to bicarbonate and proton. To date, there are twelve catalytically active human CAs, which display diverse sub-cellular localization, tissue-specific expression, and kinetic properties^2,3^. Among a broad spectrum of CA-linked research areas, clinical investigation is a major focus due to the implication of abnormal CA levels or their activities in diseases, such as glaucoma^4^, epilepsy^5^, obesity^6^, and cancer^7^. Therefore, many efforts have been dedicated over years to design CA isoform-selective compounds exhibiting sufficient affinity properties^8^. These derivatives would be prospective for the translation into the clinic because of therapeutic efficacy without inducing undesired side effects caused by inhibited vital off-target CAs. However, it is a challenging task because of the high structural homology among human CAs^9^.

CA VI is the only secreted human CA isoform found in saliva^10^, serum^11^, milk^12^, respiratory airways^13^, and alimentary canal^14^. Several studies have indicated the immunological CA VI function^15,16^ and have presented associations of CA VI with bitter taste perception^17,18^ or protection of excess acidity-caused complications, including dental caries^19,20^ and injuries of esophageal or gastric epithelium^21^. The link of CA VI with certain cancers, such as that of salivary glands, has been speculated by gene comparison study^22^, which have shown close relation of CA VI with CA IX, a marker of tumors^23^. Thus, there is a demand for effective and selective CA VI inhibitors, which would be relevant to determine the exact physiological role of CA VI.

For more than five decades, the most widely applied method in the search of CA isoform selective inhibitors has been the stopped-flow assay of the catalytic activity of CO_2_ hydration (SFA)^24,25^. However, SFA has several limitations, such as the largely unknown CO_2_ concentration and unfeasibility to measure inhibition constant below several nM^26^. Therefore, biophysical techniques, such as the fluorescent thermal shift assay (FTSA) and isothermal titration calorimetry (ITC), are promising alternatives to screen CA-targeting derivatives. FTSA is a high-throughput method exhibiting minimized biomolecule consumption and low limitations for binding affinity, thereby both strong (picomolar) and weak (millimolar) compounds can be identified during the same experiment^26–29^. ITC allows the direct determination of stoichiometry and thermodynamic parameters, such as affinity, enthalpy, entropy, and heat-capacity, during a single or several titration experiments but it demands relatively large quantities of proteins and has limitations for assessing the binding affinity^26,30,31^.

Importantly, two types of variables can be distinguished when binding reactions are carried out by FTSA or ITC: the *observed* parameters obtained from experimental setup and the *intrinsic* values calculated according to the corresponding observed data. Most studies on the development of CA inhibitors usually provide only observed binding parameters, which are dependent on experimental conditions and might be misleading. Both the CA and inhibitor exist in different protonation states in the solution compared with ones in the complex. Therefore, protonation-deprotonation reactions are required to initiate the binding of inhibitor to CA. Only intrinsic values subtract energetic contribution of binding-linked protonation events and thus are relevant for the rational drug design^32–35^.

Due to the recent advances in the structural and *in silico* biology, production of target recombinant proteins, including CAs, in large quantities is of high demand for *in vitro* inhibitor screening of drug-candidates during preclinical research. The literature lists a number of host cells for expression of recombinant proteins. Among microorganisms, the enterobacterium *Escherichia coli* (*E*. *coli*) is selected frequently owing to numerous advantages, such as rapid growth, easy genetic manipulation, and relative cost effectiveness^36,37^. However, the stability of heterologous protein in *E*. *coli* can be influenced by the several factors, including mRNA instability, codon bias, protein aggregation, toxicity, and lack of post-translational modification^38,39^. Therefore, different, more efficient strategies to obtain functionally active recombinant proteins in high yield are required for screens of chemical compounds with the aim to identify hits in the initial stages of drug discovery.

The goal of the present study was to design a CA II-based CA VI model protein, named as CA VI-mimic, for the search of CA-isoform selective inhibitors. As CA VI-mimic, mutant of CA II containing five point mutations, such as A65T, N67Q, F130Y, V134Q, L203T, was generated via site-directed mutagenesis. CA II was selected as a core for CA VI-mimic because purification yield of CA II from *E*. *coli* is ~10-fold higher than CA VI, CA II has highest catalytic efficiency among CAs, and CA II is confirmed as a stable CA protein for X-ray crystallography. Here enzymatic activity and inhibition of CA II, CAVI-mimic, and CA VI was determined by SFA. Biophysical studies on inhibitor binding to CA II, CA VI-mimic, and CA VI were carried out by ITC and FTSA. X-ray crystallography and computational modeling were used to compare positions of several inhibitors in the active sites of CA II, CA VI-mimic, and CA VI. Observed and intrinsic thermodynamics were in line with structural results which confirmed the relevance of CA VI-mimic as a CA VI model protein. The most tested benzenesulfonamides bound to CA VI-mimic in a manner corresponding to their interactions with CA VI but not CA II, thereby emphasizing suitability of the investigated CA II mutant mimicking CA VI for inhibitor screening.

## Results

### Enzymatic activity of CA VI-mimic correlates with CA II, but not CA VI

**Figure 1:**
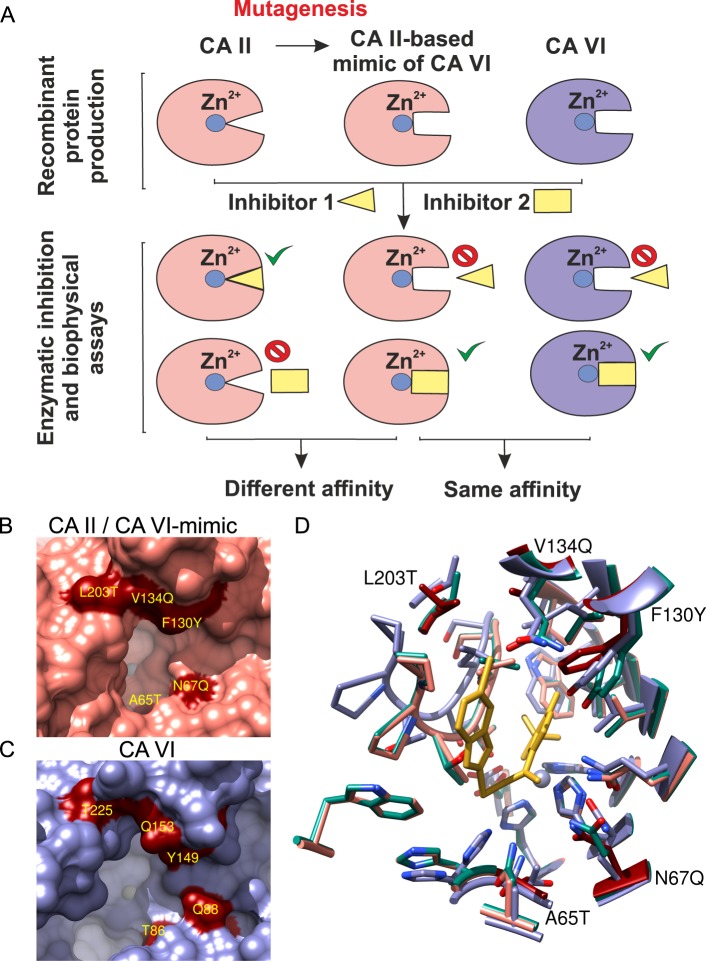
(**A**) The mimic of CA VI was prepared from CA II by site-directed mutagenesis of amino acids that differ between two CA isoforms. The CA VI-mimic protein served as a model of compound binding to CA VI. (**B**) Active site of CA II (PDB ID: 3KS3). Dark red molecular surfaces mark the positions of point mutations introduced in the active site of CA II to resemble CA VI by making a multiple-residue mutant of CA II (CA VI-mimic). (**C**) Active site of CA VI (PDB ID: 3FE4). The light blue areas are buried molecular surfaces between interacting molecules in the homodimeric complex. Dark red molecular surfaces mark the equivalent positions between multiple-residue mutant of CA II (CA VI-mimic) and CA VI. The labels belong to CA VI (CA II numbering). (**D**) Superposed structures of the binding pockets of CA II (rose; PDB ID: 3M96), CA VI (blue; PDB ID: 3FE4), and CA VI-mimic (green; PDB ID: 6QL2). The mutated residues of CA II are colored dark red. The zinc ion in the active site of each CA isoform is shown as a grayish sphere in panels (B–D).

Studies on inhibitor selectivity towards diverse human CA isoforms are important to develop efficient compounds for the treatment of diseases caused by abnormal levels or activities of a particular CA isoform. Therefore, it is essential to evaluate inhibitor affinity to all human CAs, including CA VI. Since our previous study^40^ indicated a low yield of recombinant CA VI from *E*. *coli*, we generated CA II mutant as a CA VI model protein (CA VI-mimic) for inhibitor screening. Inhibitor affinities towards CA II and CA VI-mimic were expected to differ in the way imitating inhibitor binding to CA VI, but not CA II (Fig. 1A). Thus, negligible differences between inhibitor affinities towards CA VI and CA VI-mimic were presumed. According to computational modeling, five point mutations A65T, N67Q, F130Y, V134Q, L203T were chosen (Figs 1B–D and S1) and introduced into the active site of CA II.

**Figure 2:**
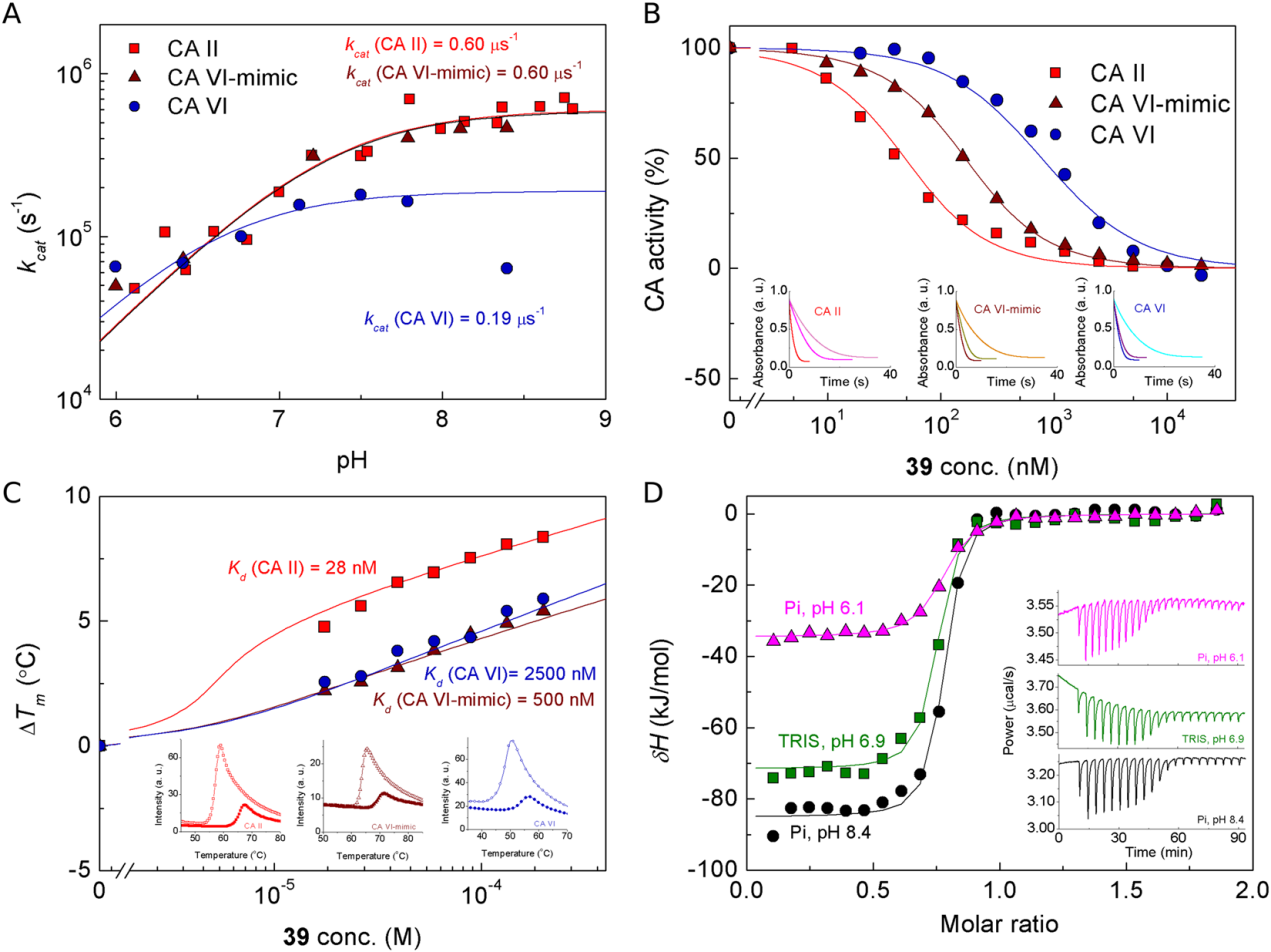
Catalytic activity, inhibition and binding profiles of CA II (red squares), CA VI-mimic (wine triangles) and CA VI (royal circles). (**A**) The plot of *k*_*cat*_ dependence on pH by stopped-flow CO_2_ hydration assay (SFA). Solid lines were fit using single protonation model. (**B**) Inhibition of CAs by compound **39** using SFA. Data points were fit to the Morrison eq. (solid lines)^74,75^. The insets show raw activity curves of CA catalyzed reaction without added inhibitor (red, wine, royal lines), CA inhibited reaction with 313 nM added compound **39** (magenta, dark yellow, purple lines) and spontaneous CO_2_ hydration reaction (pink, orange, cyan lines) in the absence of CA. (**C**) Dosing curves of compound **39** binding to CAs by fluorescent thermal shift assay (FTSA). Data points show the Δ*T*_m_ as a function of the total concentration of compound **39** added and the lines are simulated using fitting parameters when temperature is 37 °C, CA concentration is 10 µM, enthalpy of unfolding is 690 kJ/mol for CA II and CA VI-mimic, and 480 kJ/mol for CA VI, enthalpy of binding is −42 kJ/mol, heat capacity of binding is −0.8 J/(molK) and the reference melting temperature is 56.8 °C for CA II, 63.1 °C for CA VI-mimic, and 47.6 °C for CA VI. The Δ*T*_m_ shift is equal for CA VI-mimic and CA VI, but *K*_d_’s differ due to different enthalpies of unfolding. The insets show CA unfolding curves at 0 and 200 µM inhibitor **39** concentrations. (**D**) Isothermal titration calorimetry (ITC) curves of EZA binding to CA VI-mimic in phosphate (Pi, pH 6.1 (▲) and 8.4 (●)) and TRIS buffer (pH 6.9 (■)) at 25 °C. Lines were fitted using single binding site model. The insets show raw data ITC curves at 10 µM CA VI-mimic concentration. Different observed enthalpies of binding illustrate the presence of binding-linked protonation reactions that must be accounted for the determination of intrinsic binding parameters.

The catalytic activity of CA VI and CA VI-mimic to catalyze CO_2_ hydration reaction was measured by SFA (Figs 2A and S3). Analysis of kinetic data showed that site-directed mutagenesis did not significantly affect either the catalytic activity or p*K*_*a*_ of zinc-bound water molecule of CA II. Catalytic constants (*k*_*cat*_) of CA II and CA VI-mimic did not differ (*k*_*cat*_ values were 6.0 × 10^5^ s^−1^), whereas *k*_*cat*_ for CA VI was lower than CA VI-mimic by 3-fold (1.9 × 10^5^ s^−1^). In the pH range 5.9–7.0 Michaelis constants (*K*_*M*_) as well as *k*_*cat*_ values of the carbon dioxide hydration reaction were comparable: 7.3 ± 2.9 mM for CA II, 8.8 ± 1.8 mM for CA VI-mimic and 9.9 ± 3.2 mM for CA VI. However, in the pH range 7.1–8.4 *K*_*M*_ values of CA VI-mimic (6.8 ± 2.0 mM) were closer to CA II (4.7 ± 1.0 mM) than to CA VI (11.3 ± 0.7 mM). Interestingly, maximum catalytic activity of CA VI was observed at pH 7.0–8.0 and it decreased at pH above 8.0. The determined p*K*_a_ value of zinc-bound water molecule of CA VI was 6.6 ± 0.2. The observed inhibition constants by SFA correlated with dissociation constants determined by FTSA. Typical SFA curves of CA II, CA VI-mimic and CA VI inhibition by compound **39** are shown in Fig. 2B.

### Influence of buffer and pH for the observed binding affinity of ethoxzolamide to CA VI-mimic

Biophysical methods, such as ITC and FTSA, enable measurements of observed thermodynamics and thereafter calculations of intrinsic affinities. The observed binding profiles are altered by linked reactions and therefore, only intrinsic binding parameters can be correlated with compound structures, thereby revealing structural reasons for protein-ligand binding affinity.

**Figure 3:**
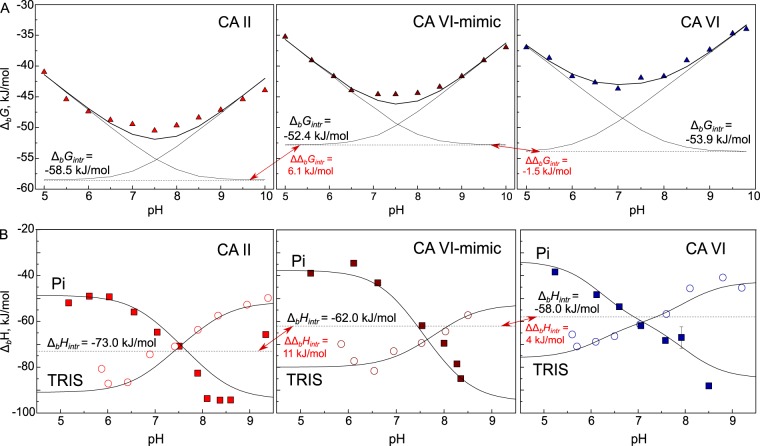
(**A**) Comparison of observed Gibbs energy changes (Δ_*b*_*G*_*obs*_) upon EZA binding to CA II (red), CA VI-mimic (dark red), and CA VI (blue) as a function of pH (25 °C). Experiments were performed by FTSA in universal buffer (50 mM sodium phosphate, 50 mM sodium acetate, and 25 mM sodium borate). The p*K*_*a*_ for CA VI-mimic was determined to be 7.1. (**B**) The observed enthalpy changes (Δ_*b*_*H*_*obs*_) upon EZA binding to CA II (red), CA VI-mimic (dark red), and CA VI (blue) as a function of pH in two different buffers (sodium phosphate (Pi) and TRIS), which have different protonation enthalpies. Experiments were performed by isothermal titration calorimetry (ITC) at 25 °C. The dashed line shows the intrinsic binding enthalpy (Δ_*b*_*H*_*intr*_), which is independent of pH. The p*K*_*a*_ for CA VI-mimic was determined to be 7.3. Thermodynamic binding parameters of EZA binding to CA II and CA VI have been previously published^40,41^. Red arrows indicate difference in Δ_*b*_*G*_*intr*_ or Δ_*b*_*H*_*intr*_ of EZA binding to CA VI-mimic compared to CA II or CA VI.

Binding energetics are significantly affected by several protonation-deprotonation events which are necessary for the binding of sulfonamide derivative to CA. Only deprotonated sulfonamides can interact with the zinc cation in the active site of pronated CA, containing zinc-coordinated water molecule (protonated hydroxy group). In this study, observed and intrinsic affinities of inhibitor binding to CA VI-mimic were determined and compared to their affinities towards CA II and CA VI. The obtained experimental data by FTSA on interactions between ethoxzolamide (EZA) and CA VI-mimic in buffers with different pH showed that pH remarkably influenced the observed binding Gibbs energy (Δ_*b*_G_*obs*_, Fig. 3A). The dependence of Δ_*b*_G_*obs*_ on pH has also been observed previously^40,41^ when EZA binding to CA II or CA VI was measured. The strongest interaction was determined near neutral pH and became weaker both in acidic and alkaline pH. Sulfonamide group usually has p*K*_*a*_ in the range between 7 and 10, whereas CA isoforms have p*K*_*a*_ around 7. Therefore, diminished EZA affinity in acidic solution was because the fraction of binding-ready deprotonated form of EZA decreased by 10-fold with every pH unit. Similarly, EZA affinity decreased in alkaline solution because the fraction of binding-ready CA with the zinc-bound protonated hydroxide (water molecule) decreased. According to U-shaped curve as the global fit of experimental data extrapolated to 25 °C, intrinsic binding Gibbs energy (Δ_*b*_G_*intr*_) change upon EZA interaction with CA VI-mimic was determined to be −52.4 kJ/mol which was 7.8 kJ/mol greater than the highest experimentally observed value (−44.6 kJ/mol at pH 7.1). Difference of Δ_*b*_G_*intr*_ (ΔΔ_*b*_G_*intr*_) between EZA interaction with CA VI-mimic and CA VI were smaller (ΔΔ_*b*_G_*intr*_ = −1.5 kJ/mol) compared to that between CA VI-mimic and CA II (ΔΔ_*b*_G_*intr*_ = 6.1 kJ/mol).

Observed standard enthalpy changes (Δ_*b*_H_*obs*_) upon EZA binding to CA VI-mimic formed an X-shaped curve which depended on pH and buffer (Fig. 3B). The same tendency has been found previously^40,41^ when Δ_*b*_H_*obs*_ of EZA binding to CA II or CA VI was analyzed. Results were obtained by ITC titration at 25 °C in two buffers exhibiting different protonation enthalpies: sodium phosphate (Pi) and TRIS. Upon EZA-CA VI-mimic titration, more than 20 kJ/mol difference in Δ_*b*_H_*obs*_ was observed in same buffer at different pHs (in TRIS buffer: −81.6 kJ/mol at pH 6.5, −57.2 kJ/mol at pH 8.5; in Pi buffer: −38.9 kJ/mol at pH 5.2, −84.9 kJ/mol at pH 8.4). To dissect protonation influence, intrinsic enthalpy (Δ_*b*_H_*intr*_) of EZA interaction with CA VI-mimic was globally fitted to be −62.0 kJ/mol. Difference of Δ_*b*_H_*intr*_ (ΔΔ_*b*_H_*intr*_) between EZA interaction with CA VI-mimic and CA VI were smaller (4.0 kJ/mol) compared to that between CA VI-mimic and CA II (11.0 kJ/mol). Thus, Δ_*b*_H_*intr*_ were in line with Δ_*b*_G_*intr*_, confirming that CA II mutant was mimicking CA VI for EZA binding.

Furthermore, analysis of U- and X-shaped curves obtained by FTSA and ITC, respectively, led to the characterization of two important parameters of CA VI-mimic: ionization constant (p*K*_*a*_) and enthalpy of protonation (Δ_*p*_*H*) of the zinc-bound water molecule (Table 1). The p*K*_*a*_ of CA VI-mimic was determined to be 7.2 at 25 °C as the average of two p*K*_*a*_ values evaluated independently by two techniques: 7.1 by FTSA and 7.3 by ITC. The p*K*_*a*_s of CA II and CA VI-mimic matched each other within the error margin of 0.2 pH unit^42^, whereas p*K*_*a*_s of CA VI and CA VI-mimic significantly differed by 1.0 pH (25 °C). Thus, target five point mutations of CA II, which were introduced to design CA VI-mimic, did not affect amino acids surrounding zinc in active sites of CA II at the level causing significant difference of p*K*_*a*_s between CA II and CA VI-mimic. Moreover, Δ_*p*_*H* for CA VI-mimic was assessed to be −38.0 kJ/mol at 25 °C. The difference of Δ_*p*_*H* between CA VI and CA VI-mimic (6.0 kJ/mol) was 2-fold lower than difference of Δ_*p*_*H* between CA II and CA VI-mimic (12.0 kJ/mol). Therefore, experiments with one inhibitor EZA resulted in both p*K*_*a*_ and Δ_*p*_*H* for CA VI-mimic, which are essential parameters to determine intrinsic energetics of any other inhibitor binding to CA VI-mimic.

**Table 1:** Thermodynamic parameters of protonation of zinc-bound hydroxide anion of studied CA isoforms as determined by FTSA and ITC at 25 °C.

Protein	p*K*_*a*_	Δ_*p*_*G*, kJ/mol	Δ_*p*_*H*, kJ/mol	TΔ_*p*_*S*, kJ/mol
CA II^a^	7.1	−40.5	−26.0	14.5
CA VI-mimic	7.2	−41.1	−38.0	3.1
CA VI^b^	6.2	−35.4	−32.0	3.4

The uncertainty of the p*K*_*a*_ values determined by FTSA and ITC is approximately 0.2 pH units, while for the change in Gibbs energies and enthalpies it is approximately 2 kJ/mol. ^a^Data taken from^41^; ^b^Data taken from^40^.

### Hydrophobic substituents and fluorine substituents significantly affected intrinsic inhibitor binding affinity for CA VI-mimic

**Figure 4:**
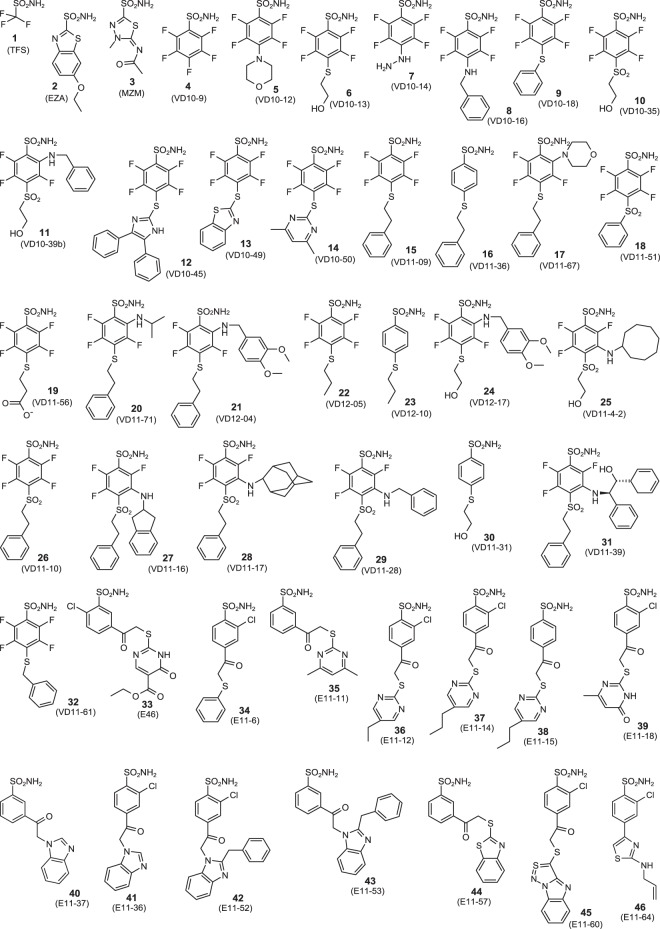
Chemical structures of **1**-**46** compounds designed as CA inhibitors. Compounds **1**-**3** are standard inhibitors of CAs that we used here as control compounds (TFS, EZA, and MZM).

Here 43 benzenesulfonamide derivatives binding to CA VI-mimic was measured by FTSA and inhibition constants of several selected compounds were confirmed by SFA. Trifluoromethanesulfonamide (TFS), EZA, and methazolamide (MZM) were used as controls. Structures of tested compounds are shown in Fig. 4, while dissociation constants (*K*_*d*_) are listed in Tables 2 and S1 (examples of raw and integrated data of inhibitor binding to CA VI-mimic by FTSA and ITC at different pHs are indicated in Fig. 2C,D, respectively).

**Table 2:** The *K*_*d*_*obs*_ and *K*_*d*_*intr*_ values (nM) for interactions between inhibitor and three CA proteins: CA II, CA VI-mimic, and CA VI.

Inhibitor	Lab. name	p*K*_*a*_SA_	*K*_*d*_ *obs*_ *(nM)*			*K*_*d*_ *intr*_ *(nM)*		
			CA II	CA VI-mimic	CA VI	CA II	CA VI-mimic	CA VI
1.	TFS	6.02	20	33	14	8.0	15	1.2
2.	EZA	7.82	1.3 (<5.0)	17 (<54)	33 (<54)	0.073	1.1	0.40
3.	MZM	6.86	100	330	830	27	97	44
4.	VD10-9	8.12	46	130	430	1.4	4.7	2.7
5.	VD10-12	8.61	18	100	1100	0.19	1.2	2.4
6.	VD10-13	8.14	11	130	200	0.32	4.2	1.2
7.	VD10-14	8.84	91	330	1000	0.57	2.4	1.3
8.	VD10-16	8.47	9.1	200	1200	0.13	3.3	3.7
9.	VD10-18	7.80	3.4	140	200	0.20	9.8	2.5
10.	VD10-35	7.28	17	130	67	2.5	22	2.1
11.	VD10-39b	7.85	83	140	130	4.6	8.8	1.5
12.	VD10-45	7.69	5.8	140	140	0.43	12	2.2
13.	VD10-49	7.83	0.65	110	200	0.037	7.2	2.3
14.	VD10-50	8.02	9.6 (<43)	330 (140)	1000 (630)	0.37	15	7.9
15.	VD11-9	8.05	1.7	100	400	0.061	4.1	3.0
16.	VD11-36	10.1	12	500	1100	0.0039	0.20	0.077
17.	VD11-67	8.67	5900	100 000	100 000	55	1000	190
18.	VD11-51	7.07	3.3	130	160	0.68	31	6.6
19.	VD11-56	7.97	20	330	500	0.86	16	4.4
20.	VD11-71	8.67	500	2500	3300	4.6	26	6.3
21.	VD12-04	8.67	1800	3300	20 000	17	35	38
22.	VD12-05	8.15	2.2	50	140	0.065	1.7	0.86
23.	VD12-10	10.2	25 (<36)	500 (200)	830 (3900)	0.0070	0.16	0.048
24.	VD12-17	8.67	1300	5000	11 000	12	52	21
25.	VD11-4-2	8.01	56	100	67	2.2	4.5	0.54
26.	VD11-10	7.22	1.2	67	140	0.21	13	4.8
27.	VD11-16	7.87	35	1000	2000	1.9	59	22
28.	VD11-17	7.87	50	140	330	2.6	8.5	3.6
29.	VD11-28	7.87	6.7	200	110	0.35	12	1.2
30.	VD11-31	9.96	140	1000	5000	0.070	0.55	0.50
31.	VD11-39	7.87	33	2000	1000	1.8	120	11
32.	VD11-61	8.53	3.3	140	330	0.042	2.1	0.87
33.	E46	8.90	50	50	200	0.28	0.31	0.23
34.	E11-6	8.70	5.6	330	1100	0.048	3.3	2.0
35.	E11-11	9.40	1000	2000	13 000	1.8	4.0	4.5
36.	E11-12	8.90	8.5	500	5000	0.047	3.1	5.7
37.	E11-14	8.90	7.1	330	3300	0.039	2.1	3.8
38.	E11-15	9.40	560	1300	5000	0.98	2.5	1.8
39.	E11-18	8.90	28 (<54)	500 (300)	2500 (1600)	0.15	3.1	2.8
40.	E11-37	9.60	3600	3300	6700	4.0	4.2	1.5
41.	E11-36	8.30	8.3	500	3300	0.16	12	14
42.	E11-52	8.70	2.9	330	1400	0.025	3.3	2.5
43.	E11-53	9.60	1700	3300	10 000	1.9	4.2	2.3
44.	E11-57	9.60	140	500	3300	0.16	0.63	0.75
45.	E11-60	8.70	250	500	3300	2.2	4.9	5.9
46.	E11-64	9.40	100	1100	17 000	0.16	2.2	5.5

The observed inhibitor affinities for CA VI-mimic were obtained experimentally by FTSA (pH 7.0, 37 °C), whereas the intrinsic parameters were calculated from the corresponding observed data using p*K*_*a*_ of 7.0 for CA VI-mimic at 37 °C as explained in the methods part. The standard error of *K*_*d*_ measurements is ±2-fold. The p*K*_*a*_ values of applied sulfonamide amino group (p*K*_*a*_SA_) and inhibitor affinities towards CA II and CA VI have been already reported^58,86^. Dissociation constants *K*_*d*_s of selected compounds were confirmed by SFA (pH 7.5). Experiments were performed at 23 °C and observed *K*_*d*_s were extrapolated to 37 °C using van’t Hoff equation when enthalpy of binding is −42 kJ/mol. The values at 37 °C are given in parentheses. The determined *K*_*d*_*s* at 23 °C are given in Table S2.

According to observed thermodynamics, EZA was shown to be the strongest binder to CA VI-mimic with observed *K*_*d*_ (*K*_*d*_*obs*_) of 17 nM. From a series of fluorinated benzenesulfonamides, compounds **22** and **26** bearing substituents at *para* position were characterized to be the most potent CA VI-mimic inhibitors bound with observed *K*_*d*_ (*K*_*d*_*obs*_) in the range of 50–67 nM. The comparison between binding affinities of corresponding fluorinated and nonfluorinated compounds (**6** vs **30**, **15** vs **16**, and **22** vs **23**) showed that fluorination significantly increased observed binding affinity and diminished p*K*_*a*_ of inhibitor sulfonamide amino group. For instance, *K*_*d*_*obs*_ for **30** and **6** binding to CA VI-mimic increased 8-fold upon fluorination (from 1000 nM to 130 nM), whereas *K*_*d*_*obs*_ for **23** and **22** increased affinity 10-fold (from 500 nM to 50 nM). Fluorines reduced p*K*_*a*_ of sulfonamide group significantly: from 9.96 to 8.14 for inhibitors **30** and **6**, respectively, and from 10.2 to 8.15 for compounds **23** and **22**, respectively (Table 2). Correspondingly, chlorine in most compounds also increased observed affinity and reduced p*K*_*a*_ of inhibitor sulfonamide amino group. For example, upon chlorination *K*_*d*_*obs*_ for **40** and **41** interaction with CA VI-mimic increased 430-fold (from 3600 nM to 8.3 nM, respectively), while p*K*_*a*_ values were lowered by 1.30 unit (from 9.60 to 8.30, respectively).

To investigate structure-activity relationships, intrinsic *K*_*d*_ (*K*_*d*_*intr*_) values for interactions between CA VI-mimic and investigated series of compounds were calculated. The largest differences between *K*_*d*_*obs*_ and *K*_*d*_*intr*_ values were determined for nonfluorinated benzenesulfonamides (**16**, **23**, and **30**), where the binding to CA VI-mimic differed 2500, 3200, and 1800-fold, respectively. Only five compounds (**10**, **18**, **26**, TFS, and MZM) exhibited lower than 10-fold difference between the *K*_*d*_*obs*_ and *K*_*d*_*intr*_. According to intrinsic thermodynamics, the strongest binders were inhibitors **16**, **23**, **30**, and **33** with *K*_*d*_*intr*_ in the range of 0.16–0.55 nM. Therefore, the strongest intrinsic interaction between inhibitor and CA VI-mimic was observed when inhibitor did not possess any fluorines in benzenesulfonamide scaffold and contained a hydrophobic substituent at *para* position, such as SCH2CH2CH3 (*K*_*d*_*intr*_ for inhibitor **23** was 0.16 nM) and SCH2CH2Ph (*K*_*d*_*intr*_ for inhibitor **16** was 0.20 nM). Exceptionally, inhibitor **33** was the strongest binder to CA VI-mimic with chlorine at *ortho* position and large hydrophilic group at *meta* position (*K*_*d*_*intr*_ was 0.31 nM). Replacement of the methyl group (inhibitor **23**) by hydrophilic hydroxyl group (inhibitor **30**) weakened intrinsic binding affinity more than 3-fold (from to 0.16 nM to 0.55 nM). Moving on to the structural analysis of fluorinated benzenesulfonamides, two inhibitors were determined to be the strongest binders to CA VI-mimic: compound **5** bearing 4-Morpholinyl group at *para* position (*K*_*d*_*intr*_ was 1.2 nM) and **22** with SCH2CH2CH3 group at *para* position (*K*_*d*_*intr*_ was 1.7 nM). In line with results obtained from nonfluorinated compounds, hydrophobic contacts between inhibitors and CA VI were identified to be significant because the exchange of methyl group (inhibitor **22**) by hydroxyl group (inhibitor **6**) or carboxyl group (inhibitor **19**) weakened intrinsic interaction by 2 and 9-fold, respectively. Apparently, the number of methyl groups of substituents at *para* position had significant effect on intrinsic binding affinity. The inhibitor **32** with SCH2Ph bound to CA VI-mimic 2-fold stronger than inhibitor **15** with SCH2CH2Ph and 4-fold stronger than inhibitor **9** with SPh. Most often, introducing diverse substituents at *meta* position did not change intrinsic binding affinity significantly (**6** vs **25**, **26** vs **28**, and **26** vs **29**), except for **26** vs **31** bearing large hydrophobic group which weakened interaction 9-fold. The compound **17** bearing two large and highly hydrophobic substituents at *ortho* and *para* positions was the weakest binder not only according to the observed parameters (*K*_*d*_*obs*_ was 100 µM), but also intrinsic data (*K*_*d*_*intr*_ of 1000 nM).

### Thermodynamically CA VI-mimic binds benzenesulfonamides similarly to CA VI but differing from CA II

**Figure 5:**
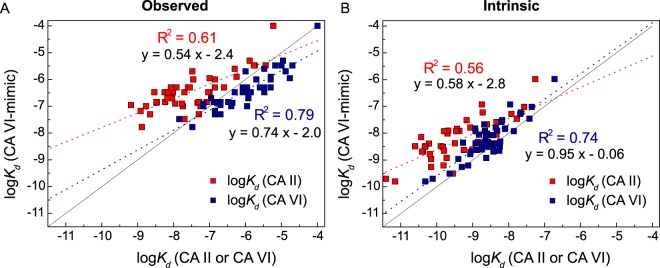
Comparison of log*K*_*d*_ values representing observed (**A**) and intrinsic (**B**) inhibitor binding affinities towards CA VI-mimic and CA II (red squares) or CA VI-mimic and CA VI (blue squares). Straight line represents a model of equal affinity of inhibitor binding to pairwise proteins. Red and blue dashed lines show linear regression models for inhibitor binding to CA II and CA VI, respectively. R^2^ values and linear equations are indicated. Experiments were performed by FTSA (pH 7.0, 37 °C).

To evaluate if CA VI-mimic based on CA II is a suitable CA VI model protein for inhibitor screening, observed and intrinsic affinities represented by logarithmic *K*_*d*_ values of inhibitor binding to CA II, CA VI-mimic, and CA VI were compared by applying linear regression. A higher linear correlation was determined between observed affinities of inhibitor binding to CA VI and CA VI-mimic (R^2^ = 0.79) compared to the observed affinities of inhibitor interaction with CA II and CA VI (R^2^ = 0.61; Fig. 5A). Analysis of the calculated intrinsic parameters were in line with experimentally measured observed data, emphasizing a stronger correlation of the intrinsic thermodynamics of inhibitor binding to CA VI and CA VI-mimic (R^2^ = 0.74) compared to that of CA II and CA VI-mimic (R^2^ = 0.56; Fig. 5B). Furthermore, regression line slopes indicating the comparison of inhibitor binding to CA VI and CA VI-mimic (0.74 for observed affinity, 0.95 for intrinsic affinity) were larger than the corresponding slopes for CA II and CA VI (0.54 for observed affinity, 0.58 for intrinsic affinity), thereby indicating a lower difference between the inhibitor binding towards CA VI-mimic and CA VI compared to CA II.

The influence of investigated CA II mutations on inhibitor binding thermodynamics was further analyzed by calculating the absolute error (AE) values from logarithmic observed or intrinsic *K*_*d*_s of inhibitor binding to CA VI-mimic, CA II, and CA VI. According to the observed thermodynamics, binding affinities of only 12 inhibitors out of 46 tested compounds towards CA VI-mimic was more similar to CA II than CA VI. For the intrinsic data, only 6 compounds were identified as CA VI-mimic binders with the affinity more alike CA II compared to CA VI. Moreover, mean absolute errors (MAEs) as the averages for each AE were also evaluated. MAEs of AE_obs,CA II_ and AE_intr,CA II_ were equal to 1.1, while MAEs of AE_obs,CA VI_ and AE_intr,CA VI_ were significantly smaller, 0.47 and 0.41, respectively. Therefore, CA VI-mimic designed via site-directed mutagenesis from CA II was characterized to be a proper model of CA VI for observed and intrinsic inhibitor binding reactions.

### Differences in inhibitor binding affinities are due different binding modes as determined by crystallographic analysis of CA II and CA VI-mimic

Despite numerous attempts, the crystal structures of recombinant CA VI complexes with sulfonamide-based inhibitors were not obtained by soaking. Even though CA VI crystals survived soaking procedure, crystals did not contain the clear electron densities of inhibitors. The co-crystallization of CA VI protein with several inhibitors failed, as we did not obtain any crystals suitable for X-ray diffraction experiment. Most likely, CA VI complexes with sulfonamide-based inhibitors cannot be crystallized using crystallization conditions that are effective for the unbound CA VI protein.

To structurally investigate the binding of benzenesulfonamides with CA VI, we have engineered CA VI-mimic and applied in crystallographic studies. We have solved crystal structures of CA VI-mimic complexes with three inhibitors (Fig. S2): EZA (PDB ID: 6QL2), inhibitor **14** (PDB ID: 6QL1), and **25** (PDB ID: 6QL3). These complexes were compared with the corresponding complexes composed of CA II and same ligands (EZA (PDB IDs: 3CAJ (X-ray), 6BCC (neutron diffraction)), inhibitor **14** (PDB ID: 4HT0), and **25** (4PYY)). The space groups and unit cell parameters of CA VI-mimic crystals were similar to those of CA II (Table 3). There was one unique protein-ligand complex in the asymmetric unit. CA VI-mimic binding pocket was found to be similar to CA VI according to crystallographic studies (Fig. 1D) followed by thermodynamic analysis. For this reason, the insights into the compound binding mode to CA VI-mimic are likely to be valid for analyzing the ligand binding data to CA VI.

**Table 3:** Data collection and refinement statistics of human CA VI-mimic and its complexes with inhibitors inhibitor **14**, **25**, and EZA.

Isoform-ligand	CA VI-mimic – inhibitor 14	CA VI-mimic - EZA	CA VI-mimic – inhibitor 25
PDB ID	6QL1	6QL2	6QL3
**Data-collection statistics**			
Space group	P12_1_1	P12_1_1	P12_1_1
Unit-cell parameters (Å)	a = 42.3, b = 41.4, c = 71.2, β = 104.3°	a = 42.1, b = 41.3, c = 71.4, β = 104.2°	a = 42.2, b = 41.4, c = 71.9, β = 104.2°
Resolution range (Å)	1.42–69.0	1.30–40.9	1.35–69.7
Wavelength (Å)	0.976300	0.975522	0.975522
Radiation source	EMBL, P14	EMBL, P14	EMBL, P14
Unique reflections number	42097	56403	52382
R_merge_, overall (outer shell)	0.042(0.241)	0.067 (0.334)	0.088 (0.338)
I/σ overall (outer shell)	22.7(7.2)	13.1 (5.0)	10.8 (4.1)
Multiplicity overall (outer shell)	7.0 (6.6)	6.9 (6.9)	6.8 (6.7)
Completeness (%) overall (outer shell)	92.8 (74.1)	96.4 (94.5)	98.8 (99.0)
Wilson B-factor	13.2	13.1	9.3
**Refinement statistics:**			
R_work_	0.157	0.119	0.116
R_free_	0.185	0.157	0.156
RMSD bond lengths, (Å)	0.011	0.013	0.033
RMSD bond angles (°)	2.000	1.991	2.195
Average B factors (Å^2^):			
all	16.7	20.4	15.0
main-chain	13.5	16.3	10.3
side-chain	16.2	21.7	14.8
inhibitors	26.9	13.3	17.3
waters	27.6	33.1	31.3
zinc	7.7	9.1	4.7
other molecules	40.5	37.0	36.9
**Number of atoms:**			
all	2562	2380	2482
protein	2181	2111	2127
inhibitor	69	16	28
water	287	226	275
zinc	1	1	1
other molecules	24	26	51
**Ramachandran statistics (%):**			
most favored regions	96	97	97
additionally allowed regions	4	3	3
outliers	0	0	0

All datasets were collected at 100 K, test set size was 10%.

**Figure 6:**
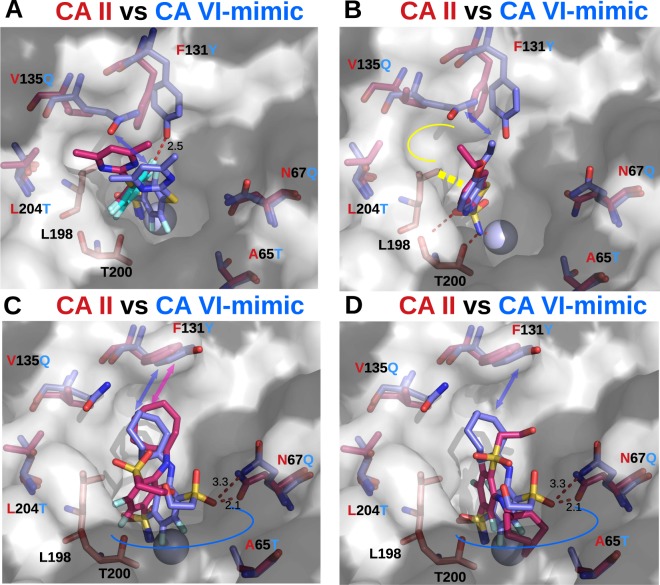
Differences in the binding structural modes of three compounds in CA II and CA VI-mimic as determined by X-ray crystallography. The zinc ion in active sites of CAs is shown as a light blue sphere. CA II side chain residues and ligands bound to CA II are colored pink and are shown transparent. CA VI-mimic side chains as well as its ligands are colored blue and also shown transparent. Mutated CA II side chains are labeled red for CA II, blue for CA VI-mimic. Hydrogen bonds are shown as dashed lines and distances are marked. Hydrophobic part of active site is shown as white surface, whereas hydrophilic part is shown as gray surface. (**A**) Compound **14** bound to active sites of CA VI-mimic (two alternative conformations of **14** are shown in cyan and blue, PDB ID: 6QL1) and CA II. The second ring of the “cyan” conformation of **14** in CA VI-mimic is not resolved in crystal structure, and not shown. (**B**) EZA bound to active site of CA VI-mimic (PDB ID: 6QL2) and CA II (PDB ID 3CAJ). Interaction of L198 with the first ring of compound is marked by thick dashed yellow line. Yellow line designates hydrophobic pocket for binding of *para*-substituent. The typical interactions for sulfonamide moiety are indicated, whereas they are omitted for clarity in other panels. (**C**, **D**) Compound **25** bound to active site of CA VI-mimic (PDB ID: 6QL3) and CA II (two alternative conformations of the ligand are indicated, PDB ID 4PYY). Blue line designates a hydrophilic part of the active site of CA VI-mimic.

The comparison of binding mode of inhibitor **14** in the active sites of CA VI-mimic and CA II is shown in Fig. 6A. Inhibitor **14** in the active site of CA VI-mimic had two alternative binding modes characterized by different positions of the fluorinated ring: the ring was either lodged between Leu198 and Thr200 side chains (colored cyan in Fig. 6A), or located in the hydrophilic part of active site (colored blue). On the other hand, in the active site of CA II we had only one position of fluorinated ring – between Leu198 and Thr200. It looks like the replacement of Phe130 in CA II with tyrosine in CA VI-mimic enabled additional position of fluorinated ring of ligand due to a steric collision between the fluorine atom of fluorinated ring and the oxygen atom of Tyr130 side chain (the close contact found in the structure was 2.5 Å). The alternative position of the fluorinated ring of compound **14** in the active site of CA VI-mimic probably was available only due to spatial fluctuations of Tyr130 side chain. Also, due to a significantly larger size and the hydrophilicity of the side chain of Gln134 in CA VI-mimic compared to Val134 in CA II, the hydrophobic dimethylpyrimidine tail of inhibitor **14** was repelled in CA VI-mimic (see *para*-group of the cyan ligand, Fig. 6A). Therefore, the change of size and the hydrophobicity/hydrophilicity of the residues 130 and 134 upon mutation could be rationalized as the main causes for the relatively significant difference in the binding affinities: inhibitor **14** bound to CA II 40-fold better than to CA VI-mimic (*K*_*d*_*intr*_ values were 0.37 nM and 15 nM for CA II and CA VI-mimic, respectively; Table 2).

The model compound EZA was bound similarly in active sites of CA II and CA VI-mimic (Fig. 6B). Some discrepancy was present only in the positions of highly flexible ethoxy moiety. The aliphatic-aromatic interactions between the methyl group of Leu198 and the first ring of EZA was present in both cases. The larger side chain of Tyr130 slightly changed the position of EZA aromatic ring in CA VI-mimic as compared with CA II due to steric conflicts. It is important also to note the role of the residue 134 interacting with the hydrophobic tail of EZA, similarly to the observation for inhibitor **14** above. The hydrophobic Val134 sidechain in CA II was mutated into hydrophilic Gln134 in CA VI, leading to the worsening of the interaction. Thus, the mutations of residues 130 and 134 were the likely reasons for 15-fold stronger binding of EZA to CA II, as determined by intrinsic thermodynamics (*K*_*d*_*intr*_ values were 0.073 nM and 1.1 nM for CA II and CA VI-mimic, respectively; Table 2).

The intrinsic binding parameters of inhibitor **25** towards CA II and CA VI-mimic were comparable (2.2 nM vs 4.5 nM, respectively; Table 2). In contrast, the binding modes of the compound found in crystal structures were different in these active sites. In CA II, inhibitor **25** had two alternative conformations: (1) the fluorinated ring located between Leu198 and Thr200, whereas the cyclooctyl ring replaced the side chain of Phe130 (Fig. 6C, pink ligand); (2) the fluorinated ring positioned in the hydrophobic part of active site, while the cyclooctyl ring – in the hydrophilic part (Fig. 6D, pink ligand). In CA VI-mimic, compound **25** had one well-defined conformation (Fig. 6C, D, blue) in which the cyclooctyl ring replaced the Tyr130 side chain, whereas the fluorinated ring occupied the hydrophilic part of active site. We can explain the presence of the single conformation of inhibitor **25** bound to CA VI-mimic. It seems that the mutation in position 67 (asparagine to glutamine) allows the position of fluorinated ring in hydrophilic part of active site when the *para*-substituent of ligand does not have sterical collision with side chain of residue 67 (compare side chain conformations of asparagine (CA II) and glutamine (CA VI-mimic), Fig. 6C, D). The same position of glutamine is found in the complexes of CA VI-mimic with inhibitor **14** and EZA ligands which means that compound **25** does not influence the position of side chain of residue 67 in CA VI-mimic. The replacement of asparagine (CA II) to glutamine (CA VI-mimic) creates the additional free space in the active site and allows for another binding mode.

## Discussion

Nowadays enzymes encompass over one-third of drug targets investigated by large pharmaceutical companies^43^, thereby emphasizing the relevance of target-based drug approach. This strategy aims to identify the compounds which would exhibit the most therapeutically beneficial effect via modulating catalytic activity or expression levels of disease-associated enzymes. The present study is focused on CA VI isoform as a drug target due to the link of CA VI with several pathologies^19,20,22^. Even though several studies on the design of compounds targeting CA VI have been reported^40,44,45^, CA VI-selective inhibitor has not been discovered so far. Therefore, there is an interest in inhibitors with high affinity and selectivity against CA VI which would be crucial to reveal biological function of CA VI.

During preclinical development, numerous high-throughput screening assays are employed to design and optimize hits toward a target protein. Therefore, *in vitro* techniques require high quantities of recombinant proteins for the proper evaluation of compound quality, efficacy, and safety before testing in humans. Despite recent advances in molecular sciences, difficulties in the production of recombinant proteins in large scale are observed. Our previous study^40^ indicated low yield of CA VI from *E*. *coli*. Here we have presented a strategy to apply CA VI-mimic as CA VI model protein for the investigation on enzymatic inhibition and inhibitor binding thermodynamics. The CA VI-mimic was designed via site-directed mutagenesis from CA II by introducing five point mutations, such as A65T, N67Q, F130Y, V134Q, and L203T. Such approach to obtain CA protein based on the CA II mutant for the search of CA-selective inhibitors has been successfully applied previously. It was due to troubles to obtain sufficient amounts of recombinant CA isoforms and enabled by high structural homology between human CAs. McKenna’s group reported a number of structural studies on CA IX-mimic based on CA II mutant with 2 mutations (S65A, Q67N)^46–48^, 7 mutations (A65S, N67Q, E69T, I91L, F131V, K170E, L204A)^49–51^ or 8 mutations (A9K, S65A, Q67N, T69E, L91I, V131F, E170K, A204L)^52^. Our group also published the exploration on inhibition parameters and binding thermodynamics via the application of CA IX-mimic as CA II mutant with 6 mutations (S65A, Q67N, L91I, V130F, L134V, A203L) and CA XII-mimic as CA II mutant with 6 mutations (S65A, K67N, T91I, A130F, S134V, N203L)^53^. The significant findings of the listed studies promoted the present investigation of CA II mutant mimicking CA VI. Over 10-fold higher purification yield of CA VI-mimic compared to CA VI from *E*. *coli* allowed kinetic, thermodynamic, and structural analyses of 43 benzenesulfonamides binding to CA VI-mimic. Even though the most tested inhibitors exhibited moderate affinities towards CA VI-mimic, this study provided insight into the structure-based design of inhibitors with better affinity and selectivity towards CA VI.

The observed kinetic parameters of CA VI and CA VI-mimic are consistent with previous works. The determined catalytic constant of CA VI compares reasonable well to published *k*_cat_ value (3.4 × 10^5^ s^−1^)^44^. The difference of *k*_cat_ values most likely arise from the uncertain CO_2_ concentration in the previous work. The CA VI-mimic had the same catalytic activity as CA II (*k*_cat_ – 6.0 × 10^5^ s^−1^) and confirmed previously published results that A65, N67, F130, V134, L203 amino acids in the active site of CA II are not important for catalytic activity^54–56^. Three times higher catalytic activity of CA VI-mimic than CA VI is an advantage in measuring nanomolar inhibition constants by SFA, because similar to all enzymatic methods it is limited by both CA activity and concentration^26^.

The relevance of protonation-deprotonation reactions occurring additionally upon inhibitor binding to CA has been reviewed by several groups^57,58^. Such protonation events have been recently confirmed by neutron crystallography^59,60^. To generate compounds with great affinities by rational design, it is essential to understand the structural reasons for the changes in binding affinities of the investigated compounds towards the target. Only intrinsic parameters subtract the contribution of protonation reactions occurring in conjunction with the binding reaction between the CA and inhibitor. In the present study, nonfluorinated benzenesulfonamides exhibited stronger intrinsic and weaker observed binding affinity than corresponding fluorinated compounds. This result is in line with the previous investigation^34^, emphasizing the impact of fluorine electronegativity on the lowering of the p*K*_*a*_ of inhibitor sulfonamide group but not the direct recognition of CA VI-mimic surface. The diminished p*K*_*a*_ of fluorinated inhibitors led to the elevated observed affinity due to the increased fraction of inhibitor in the deprotonated form that bound to CA VI-mimic with the protonated zinc-bound hydroxide ion in the active site. Furthermore, substituents at *ortho* and *para*, but not *meta* positions were identified to be significant for the molecular recognition between the compound and CA VI. However, *ortho* and *meta*-substituted benzenesulfonamides have been recently shown to act as tight CA IX binders^32^. Therefore, such findings confirmed that intrinsic, but not observed parameters should be applied to analyze the dependence of binding efficiency on compound chemical structures, thereby allowing important structure-thermodynamics correlations to design CA-isoform selective inhibitors.

Inhibitor binding affinity can be significantly affected by structural properties of CA VI which have been widely investigated. The crystal structure of recombinant CA VI catalytic domain, lacking signal sequence and C-terminal region, has revealed its dimeric arrangement with the active sites of monomers facing each other and directed towards the center of the dimer^61^. Interestingly, the recent study^62^ has indicated that pentraxin domain (PTX) is present in non-mammalian CA VI, whereas PTX-coding exon is not found in mammalian *CA6* gene most likely due to rearrangements occurring upon the duplication of the adjacent glucose transporter genes. Instead of the PTX domain, mammalian CA VI contains a C-terminal region of at least 25 residues which is not detected in other vertebrate CA isoforms. This part of CA VI may be important to form oligomers and bind other proteins affecting CA VI enzymatic activity or causing biological effect as a consequence of CA VI-protein interactions. Therefore, *in vitro* and *in vivo* studies on targeting CA VI can yield discrepancies in results because of the structural differences between recombinant CA VI applied for inhibitor screening and endogenous CA VI of live model organisms, such as mice or zebrafish.

The combination of data obtained from enzymatic inhibition and biophysical binding methods, such as FTSA and ITC, significantly strengthens the conclusions of compound structure-activity relationships. Since techniques are based on different strategies to characterize inhibitor efficacies, the precision and accuracy of the measurements are necessary to reliably select the most potent and strongest inhibitors/binders. The uncertainty and repeatability of FTSA^42^ and ITC^63,64^ measurements have been previously discussed. Furthermore, correlation between the affinities determined by FTSA and SFA or FTSA and ITC have been recently reported^26,58^ and confirmed in the present study. Therefore, both enzymatic inhibition and biophysical binding techniques are necessary for precise identification of inhibitors with great affinity and selectivity towards the particular CA isoform, thereby leading to the success in clinical development.

In conclusion, this study on site-directed mutagenesis of residues in the active site of CA II to resemble CA VI gave clues to the basis for isoform specificity of benzenesulfonamides towards CA VI over CA II. The characterization of numerous properties, such as kinetics of binding, inhibition profiles and the mechanism of action, provided the deeper insight into the efficacy of CA VI-targeting inhibitors. This *in vitro* step is crucial because only sufficiently characterized compound can result in the success on translating experimental data to a clinical disease setting. Moreover, the present kinetic, thermodynamic, and structural information is important for experiments *in silico*, such as machine learning, when current binding information will be determined, filtered, and extracted.

## Methods

### Synthesis of CA inhibitors

The synthesis of CA inhibitors has been previously described^65–68^. EZA, MZM and TFS were purchased from Sigma-Aldrich (St. Louis, MO, USA) and were used without further purification.

### Production of CA VI-mimic protein

The structural superpositions of proteins for Figs 1D and 6(A–D) were performed using UCSF Chimera v. 1.12^69^. The residues within 5 Å from the typical CA II inhibitor in PDB entry 3M96 in both isoforms were analyzed, and five residues which were different between the isoforms were selected to create CA VI-mimic. The structure-based alignment was generated using PROMALS3D web server^70^. The sequence alignment figure was prepared using TeXshade package^71^.

The expression vector pET15b-CA II^72^, encoding full length CA II (1-260), was used in site-directed mutagenesis. The residues located in CA II active site, A65, N67, F130, V134, and L203, were replaced to T, Q, Y, Q, and T, respectively. For each mutagenesis reaction two oligonucleotide primers (sense and antisense) with target mutation were used: **A65T_s**: 5‘CTC AAC AAT GGT CAT ACT TTC AAC GTG GAG3’ and **A65T_a**: CTC CAC GTT GAA AGT ATG ACC ATT GTT GAG; **N67Q_s**: CAA TGG TCA TAC TTT CCA GGT GGA GTT TGA TGA C and **N67Q_a**: GTC ATC AAA CTC CAC CTG GAA AGT ATG ACC ATT G; **F130Y_s**: CCA AAT ATG GGG ATT ATG GGA AAG CTG TGC AG and **F130Y_a**: CTG CAC AGC TTT CCC ATA ATC CCC ATA TTT GG; **V134Q_s**: GAT TAT GGG AAA GCT CAG CAG CAA CCT GAT GG and **V134Q_a**: CCA TCA GGT TGC TGC TGA GCT TTC CCA TAA TC; **L203T_s**: GAC CAC CCC TCC TCT TAC GGA ATG TGT GAC CTG and **L203T_a**: CAG GTC ACA CAT TCC GTA AGA GGA GGG GTG GTC. PCR was carried out with high fidelity *Pfu* DNA polymerase (Thermo Fisher Scientific), except V134Q created with Phusion DNA polymerase (Thermo Fisher Scientific). Composition of PCR: 1× polymerase buffer, 50 ng template DNA, 0.2 mM dNTPmix, 125 ng each sense and antisense primer, and 1.5 U DNA polymerase. Thermal cycling conditions: initial denaturation – 95 °C for 10 min, then 18 cycles: 95 °C for 3 min, annealing – 66 °C (A65T and F130Y), 63 °C (N67Q), 71 °C (V134Q), or 72 °C (L203T) for 2 min, extension – 72 °C for 8 min, final extension – one time 72 °C for 10 min. After temperature cycling, PCR product was treated with *Dpn I* restriction endonuclease in order to digest the parental DNA template and to select new synthesized mutated DNA^73^. The mutations were confirmed by DNA sequencing.

Expression of CA VI-mimic protein was carried out in *E*. *coli* BL21(DE3) strain. Transformed cells colony was transferred to LB medium, containing 100 µg/ml ampicillin, grown at 37 °C and 220 rpm for 16 h. Then the saturated culture was diluted (1:50) in fresh LB medium, containing 100 µg/ml ampicillin and 60 µM ZnSO_4_ and grown to OD_600_ ≈ 0.8. The expression of CA VI-mimic protein was induced with 0.2 mM isopropyl-β-D-thiogalactoside (IPTG) and 0.4 mM ZnSO_4_. The cells were grown over night at 19 °C, 220 rpm and harvested by centrifugation at 4000 g for 20 min at 4 °C.

The biomass was suspended in the lysis buffer (20 mM HEPES, 0.15 M NaCl, and 1 mM PMSF, pH 7.4), incubated at 4 °C for 60 min and then disrupted by sonication. Debris of cells and insoluble proteins precipitated after centrifugation at 30 000 g for 25 min. The soluble CA VI-mimic protein was purified using a metal chelate and CA-affinity chromatography. For the metal chelate chromatography the column was equilibrated with 20 mM HEPES, 0.15 M NaCl (pH 7.4). For elution of CA VI-mimic protein, solution composed of 20 mM HEPES, 0.15 M NaCl, and 0.2 M imidazole (pH 7.4) was used. Eluted protein was purified using a CA-affinity column containing *p*-aminomethylbenzene sulfonamide-agarose (Sigma-Life Science Aldrich). Sorbent was equilibrated with 20 mM HEPES, 0.15 M NaCl (pH 7.4). For the protein elution, solution composed of 0.1 M sodium acetate and 0.5 M sodium perchlorate (pH 5.6) was used. Eluted CA VI-mimic protein was dialyzed into storage buffer containing 20 mM HEPES, 0.05 M NaCl, pH 7.4, and stored at −80 °C.

The purity of CA VI-mimic protein was analyzed by SDS-PAGE. Protein concentrations were determined by UV-vis spectrophotometry using extinction coefficient ɛ_280_ = 51910 M^−1^ cm^−1^ and confirmed by standard Bradford method. Molecular mass of CA VI-mimic protein was confirmed by Mass spectrometer: observed – 29192.4 Da, theoretically predicted – 29323.0 Da. The difference is due to Met residue removed during production.

### Enzymatic activity and inhibition by SFA

Enzymatic activity and inhibition experiments were performed using an Applied Photophysics SX.18MV-R stopped-flow spectrophotometer at 23 °C. Saturated CO_2_ solution was prepared by bubbling the CO_2_ gas in Milli-Q water at 23 °C for 1 h. The concentration of CO_2_ was determined using a model described previously^26^.

Catalytic constants *k*_cat_ and Michaelis constants *K*_M_ of CA VI and CA VI-mimic were determined in a pH range from 6.0 to 8.4 using 25 mM buffer and 30–300 µM indicator systems with similar p*K*_*a*_ values: MES (p*K*_*a*_ 6.1) and Bromocresol Purple (p*K*_*a*_ 6.4, λ - 590 nm, pH 6.0–6.4), MOPS (p*K*_*a*_ 7.2) and Bromothymol Blue (p*K*_*a*_ 7.1, λ - 615 nm, pH 6.8–7.1), HEPES (p*K*_*a*_ 7.5) and Phenol Red (p*K*_*a*_ 7.5, λ - 557 nm, pH 7.2–7.8), TRIS (p*K*_*a*_ 8.06) and *m*-Cresol Purple (p*K*_*a*_ 8.3, λ - 575 nm, pH 8.0–8.4). CA VI and CA VI -mimic concentration was 50–100 nM. The ionic strength of solution was maintained at 0.2 M by the addition of sodium sulfate. Maximal velocities *v*_max_ were obtained using Lineweaver-Burk coordinates, and *k*_cat_, p*K*_a_ values were determined using single ionization model:

$${k}_{cat}=\frac{{k}_{cat-max}}{1+{10}^{pH-p{K}_{a}}}$$

Enzyme inhibition experiments were performed using 25 mM HEPES buffer containing 0.2 M sodium sulfate and 50 µM Phenol Red indicator, pH 7.5. Enzyme concentration was 10–30 nM for CA II, and 50 nM for CA VI and 50–107 nM CA VI-mimic. Inhibitor concentration was 0–20 µM in <0.2% DMSO. Raw curves were fitted using a single exponential model and the inhibition constants were determined using Morrison equation^74,75^:

$$CA\,act.( \% )=1-\tfrac{([CA]+[I]+I{C}_{50}-\sqrt{{([CA]+[I]+I{C}_{50})}^{2}-4[CA][I])}}{2[CA]}\cdot 100 \% $$

where [CA] is the total concentration of the active CA molecules, [I] is the total added inhibitor concentration, and *IC*_50_ is the concentration of inhibitor that achieves 50% inhibition of enzymatic activity. A dose-response curve was fitted using fixed CA concentration and assuming that it is equal to the active enzyme concentration.

### Inhibitor binding by FTSA

FTSA measurements were performed using a Corbett Rotor-Gene 6000 (Qiagen Rotor-Gene Q) instrument using the blue channel (excitation 365 ± 20 nm, detection 460 ± 15 nm). Samples contained 10 µL of 10 µM CA VI-mimic protein, 10 µL of 0–200 µM inhibitor in 50 mM phosphate buffer at pH 7.0 containing 100 mM NaCl, 50 µM solvatochromic dye 8-anilino-1-naphthalene sulfonate (ANS) and a final DMSO concentration of 2%. The applied heating rate was 1 °C/min. The pH dependence of the observed binding constant was measured in universal buffer containing 50 mM sodium phosphate, 50 mM sodium acetate, 25 mM sodium borate at pH 5.0–10.0. Data were fitted and analyzed as previously described^72,76^. Experiments were repeated at least twice.

### Inhibitor binding by ITC

ITC measurements were performed using a VP-ITC instrument (Microcal Inc., Northampton, USA) with 1.4 mL of 4–6 µM CA VI-mimic protein solution in the cell and 300 µL of 40–60 µM ligand solution in the syringe. A typical experiment consisted of 25–30 injections (10 µL each) added at 200–240 s intervals. In order to determine the pH dependence of the observed binding enthalpy, experiments were performed at 25 °C in 50 mM phosphate or 50 mM TRIS buffer containing 100 mM NaCl at pH 5.0–10.0 with a final DMSO concentration of 1%, equal in the syringe and the cell. Data were integrated, fitted and analyzed as previously described^77^. Experiments were repeated at least twice.

### Calculation of the intrinsic thermodynamics

The enzymatic or biophysical assays allow the determination of *observed* inhibitor affinity for CA. However, observed parameters depend on buffer or pH. Therefore, observed values are only relevant for the comparison of inhibitor binding affinities towards the target using the same experimental conditions and should not be used in structure-thermodynamics correlations in drug design.

Several protonation events take place upon the interaction between the inhibitor and CA: protonation of zinc-bound hydroxide in the active site of CA, deprotonation of inhibitor sulfonamide group, bond formation between CA and inhibitor, and compensating protonation-deprotonation reactions of buffer. To develop compounds with great affinities in the rational drug design, *intrinsic* parameters must be determined by subtracting the contribution of protonation reactions occurring in the conjunction with the inhibitor binding to CA^58^.

The parameter of *K*_*d*_*intr*_ is directly related to *K*_*d*_*obs*_ and fractions of deprotonated sulfonamide-based inhibitor $({f}_{RS{O}_{2}N{H}^{-}})$ and CA with protonated zinc-bound hydroxide (water molecule) in the active site $({f}_{CAZn{H}_{2}O})$.

$${K}_{d\_intr}={K}_{d\_obs}\times {f}_{RS{O}_{2}N{H}^{-}}\times {f}_{CAZn{H}_{2}O}$$

The fractions of binding-ready inhibitor and CA depend on the p*K*_*a*_ of sulfonamide amino group (p*K*_*a*_SA_) and the p*K*_*a*_ of water molecule in the active site of CA (p*K*_*a*_CA_), respectively.

$${f}_{RS{O}_{2}N{H}^{-}}=\frac{{10}^{pH-p{K}_{a\_SA}}}{1+{10}^{pH-p{K}_{a\_SA}}}$$

$${f}_{CAZn{H}_{2}O}=1-\frac{{10}^{pH-p{K}_{a\_CA}}}{1+{10}^{pH-p{K}_{a\_CA}}}$$

The intrinsic Gibbs energy change (${{\rm{\Delta }}}_{b}{G}_{intr}$) is associated with the change in *K*_*d*_*intr*_ for the binding reaction.

$${{\rm{\Delta }}}_{b}{G}_{intr}=RT\,\mathrm{ln}\,{K}_{d\_intr}$$

The *K*_*d*_*intr*_ values for the tested benzenesulfonamide binding to CA VI-mimic were calculated using the p*K*_*a*_ of 7.0 for CA VI-mimic at 37 °C.

The ${{\rm{\Delta }}}_{b}{H}_{obs}$ was measured by ITC as the sum of enthalpies caused by inhibitor binding to CA and protonation events, such as protonation enthalpies of buffer (${{\rm{\Delta }}}_{p}{H}_{buf}$), sulfonamide inhibitor (${{\rm{\Delta }}}_{p}{H}_{SA}$), and hydroxide bound to zinc in the active site of CA (${{\rm{\Delta }}}_{p}{H}_{CA}$):

$${{\rm{\Delta }}}_{b}{H}_{intr}={{\rm{\Delta }}}_{b}{H}_{obs}-{n}_{SA}{{\rm{\Delta }}}_{p}{H}_{SA}-{n}_{CA}{{\rm{\Delta }}}_{p}{H}_{CA}+{n}_{buf}{{\rm{\Delta }}}_{p}{H}_{buf},$$

where ${n}_{SA}={f}_{RS{O}_{2}N{H}^{-}}-1$ is the number of protons released from the inhibitor to buffer, ${n}_{CA}=1-{f}_{CAZn{H}_{2}O}$ is the number of protons bound to zinc-bound hydroxide of CA, and ${n}_{buf}={n}_{CA}+{n}_{SA}$ is the sum of uptaken or released protons by buffer. The enthalpy of protonation of TRIS and sodium phosphate buffers at 25 °C is equal to −47.4 kJ/mol and −5.1 kJ/mol, respectively^78^.

### Crystallization

The CA VI-mimic was concentrated by ultrafiltration to 19 mg/mL. Crystallization condition (buffer) was 0.1 M sodium BICINE (pH 9.0), 0.2 M ammonium sulfate and 2 M sodium malonate (pH 7.0). The ligand solutions for crystal soaking were made by mixing of 50 μL of corresponding reservoir solution and 1 μL of 50 mM ligand solution (in DMSO).

### Data collection and crystallographic structure determination

Three datasets of X-ray diffraction (CA VI-mimic in complex with inhibitor **14** (VD10-50), **25** (VD11-4-2), and EZA) were collected at the EMBL beamline P14. The datasets were processed by XDS program^79^. The molecular replacement was made by MOLREP program^80^ using as initial model 4HT0. The 3D models of compounds were created by AVOGADRO program^81^. The library files which contain complete chemical and geometric descriptions of compounds were created using LIBCHECK program^82,83^. The models were prepared using COOT^84^ and refined using REFMAC^85^. All represented graphics were made using Pymol programs (PyMOL, version 1.8.4.0). Coordinates and structure factors have been deposited to the RCSB Protein Data Bank (PDB). The PDB access codes are listed in Table 3.

## Supplementary information

Supplementary
