# Supplementary material for: Engineered Carbonic Anhydrase VI-Mimic Enzyme Switched the Structure and Affinities of Inhibitors
Source: Sci Rep. 2019 Sep 3;9:12710. doi: 10.1038/s41598-019-49094-0 (PMC6722136; doi:10.1038/s41598-019-49094-0)
Supplement: Supplementary file 1 — Supplementary [file 41598_2019_49094_MOESM1_ESM.pdf]

Engineered Carbonic Anhydrase VI-Mimic Enzyme Switched the Structure and Affinities of Inhibitors

Justina Kazokaitė[a], *Visvaldas Kairys*[b], *Joana Smirnovienė*[a], *Alexey Smirnov*[a], *Elena Manakova*[c], *Martti Tolvanen*[d], *Seppo Parkkila*[e], *and Daumantas Matulis*[a]

[a] - Department of Biothermodynamics and Drug Design, Institute of Biotechnology, Vilnius University, Saulėtekio 7, Vilnius LT-10257, Lithuania
[b] - Department of Bioinformatics, Institute of Biotechnology, Vilnius University, Saulėtekio 7, Vilnius LT-10257, Lithuania.
[c] - Department of Protein-DNA Interactions, Institute of Biotechnology, Vilnius University, Saulėtekio 7, Vilnius LT-10257, Lithuania
d Department of Information Technology, University of Turku, FI-20520, Turku, Finland
e Tampere University, Faculty of Medicine and Health Technology; Fimlab Ltd, Tampere University Hospital, Arvo Ylpön katu 34, FI-33520 Tampere, Finland

Corresponding author: Justina Kazokaitė
Department of Biothermodynamics and Drug Design, Institute of Biotechnology,
Vilnius University, Saulėtekio 7, Vilnius LT-10257, Lithuania
Tel: +37062490902
Email: kazokaite@ibt.lt

Supplementary

Figure S1. The structural alignment of CA II and CA VI sequences (UniProt codes CAH2_HUMAN and CAH6_HUMAN). The residues in the binding site and the mutated amino acids of CA II to design CA VI-mimic protein are indicated underneath the sequences. The binding site residues shown here correspond to the residues shown in Figure 1D.

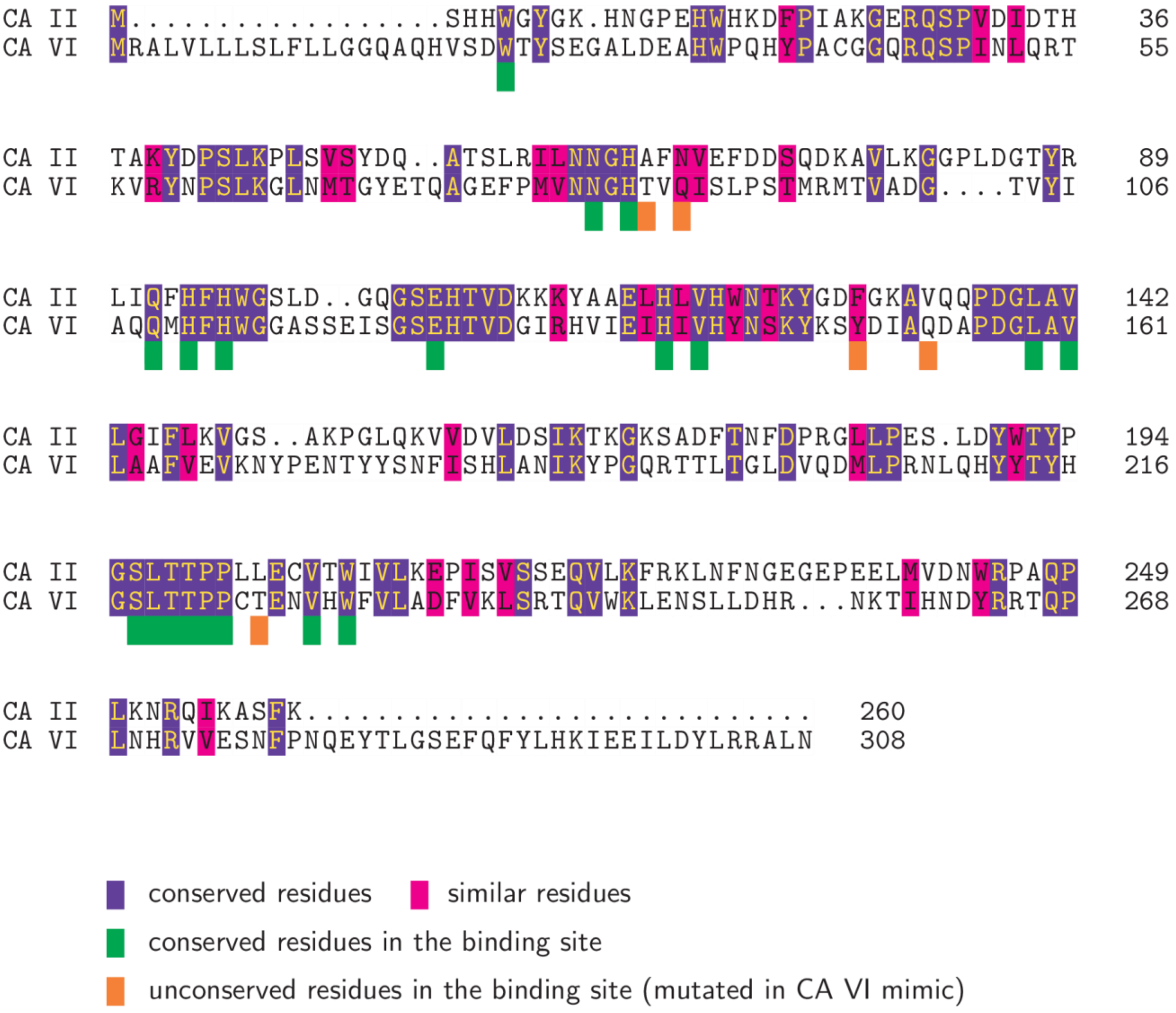

Figure S2. Crystal structures described in this study. The electron density |F(o)-F(c)| of the ligands is calculated in the absence of ligand and contoured at 3σ. Zinc ion is shown as blue sphere. (A) Compound EZA bound to CA VI-mimic (PDB ID 6QL2). (B) Compound **14** bound to CA VI-mimic (PDB ID 6QL1). (C) Compound **25** bound to CA VI-mimic (PDB ID 6QL3).

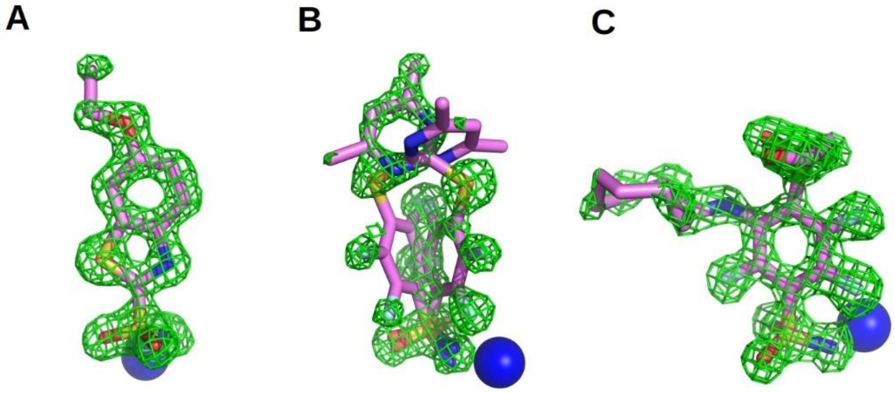

Figure S3. The plot of K_M dependence on pH for CA II (red squares), CA VI-mimic (wine triangles) and CA VI (royal circles).

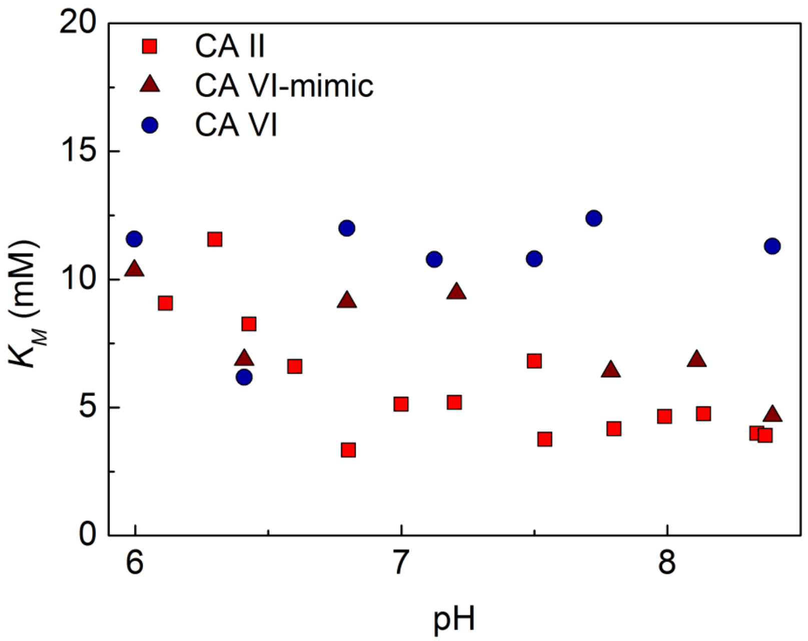

Table S1. Dissociation constants K_ds of selected compounds determined by SFA at pH 7.5, 23 ºC.

**Inhibitor**	**Lab. name**	**pK_{a_SA}**	***K_{d_obs} (nM)***		
			**CA II**	**CA VI-mimic**	**CA VI**
**2.**	EZA	7.82	<2.5	<25	<25
**14.**	VD10-50	8.02	<20	67	290
**23.**	VD12-10	10.2	<17	91	1800
**39.**	E11-18	8.90	<25	140	750
